
JOURNAL INFORMATION
==============================
NLM Title Abbreviation: Pregnancy (Hoboken)
Journal ID: Pregnancy (Hoboken)
NLM Journal ID: 9918994890806676
Journal ID: PMF2

Pregnancy

EISSN: 2997-9684
Publisher: Wiley

ARTICLE INFORMATION
==============================
PMCID: PMC13344649
DOI: 10.1002/pmf2.70162
Article ID: PMF270162
Article version: 1
Subjects: Abstracts, Oral Concurrent Session 4

oral concurrent session 4 Equity, Public Health, and Public Policy

Abstracts 45 – 53 THURSDAY February 12, 2026 1 PM – 3:15 PM Summit 201 – 204 moderators Terri‐Ann Bennett, MD Bronwen Kahn, MD

Electronic publication date: 2026 Feb 9
Publication date: 2026 Jan
Volume: 2
Issue: Suppl 1
Electronic Location ID: e70162
Accepted 2025 Oct 16
Copyright: © 2026 Society for Maternal‐Fetal Medicine. Pregnancy published by Wiley Periodicals LLC on behalf of the Society for Maternal‐Fetal Medicine.
License: This is an open access article under the terms of the http://creativecommons.org/licenses/by/4.0/ License, which permits use, distribution and reproduction in any medium, provided the original work is properly cited.
License URL: https://creativecommons.org/licenses/by/4.0/

Figure count: 0
Table count: 0
Page count: 1

source-schema-version-number: 2.0
cover-date: January 2026
details-of-publishers-convertor: Converter:WILEY_ML3GV2_TO_JATSPMC version:6.8.3 mode:remove_FC converted:07.07.2026

==============================

### Oral Concurrent Session 4

### Equity, Public Health, and Public Policy

Abstracts 45 – 53

THURSDAY
February 12, 2026
1 PM – 3:15 PM
Summit 201 – 204

MODERATORS
Terri-Ann Bennett, MD
Bronwen Kahn, MD
